# Advancements and Applications of Focused Assessed Transthoracic Echocardiography: A Narrative Review

**DOI:** 10.1002/hsr2.71868

**Published:** 2026-05-13

**Authors:** Habib Olatunji Alagbo, Oluwaremilekun Zeth Tolu‐Akinnawo, Isaac Adewumi Babawale, Charles Oluwarotimi Poluyi, Selimat Ibrahim, Jonas Lotanna Ibekwe, Dania Jaamour, Toluwalase Awoyemi

**Affiliations:** ^1^ Faculty of Medicine University of Coimbra Coimbra Portugal; ^2^ Meharry Medical College Nashville Tennessee USA; ^3^ College of Medicine University of Ibadan Ibadan Nigeria; ^4^ Brookdale University Hospital and Medical Centre New York City New York USA; ^5^ Center for Malaria and Other Tropical Diseases Ilorin Nigeria; ^6^ Feinberg School of Medicine Northwestern University Chicago Illinois USA

**Keywords:** bedside ultrasound, clinical applications, FATE, Focused Assessed Transthoracic Echocardiography, narrative review

## Abstract

**Background and Aims:**

Focused Assessed Transthoracic Echocardiography (FATE), developed by Dr. Erik Sloth in 1989, has become a key ultrasound technique for rapid bedside assessment of cardiac function. Its non‐invasive nature, real‐time imaging, and portability make it ideal for facilitating swift clinical decisions across various specialties. This review aims to explore the evolution of FATE, evaluate its current clinical applications, and discuss prospects, with a focus on training protocols, image interpretation, and the incorporation of emerging technologies.

**Methods:**

A narrative review of the literature was conducted, examining studies and guidelines related to the development, methodology, and implementation of the FATE protocol. Emphasis was placed on evidence regarding standard imaging views, training practices, and the integration of new technologies in clinical settings.

**Results:**

The FATE protocol utilizes standard echocardiographic views to enable the rapid detection of critical cardiac conditions such as cardiac tamponade, valvular dysfunction, and left ventricular failure. Its ease of use and effectiveness have led to widespread adoption in diverse settings, including resource‐limited environments and by non‐cardiologists. The review highlights improvements in training and image acquisition techniques while also noting challenges such as operator variability and the need for ongoing quality assurance. Emerging technologies, notably artificial intelligence and handheld ultrasound devices are beginning to expand FATE's diagnostic capabilities.

**Conclusion:**

FATE is a versatile and effective tool for rapid cardiac assessment that continues to evolve with advancements in technology and training. Addressing challenges such as operator variability will be essential for maximizing its clinical utility. Future research should focus on refining these aspects to further enhance patient outcomes.

**Abbreviations**
A4C: Apical Four‐Chamber
ACEP: American College of Emergency Physicians (ACEP)
AI: Artificial intelligence
ASE: American Society of Echocardiography (ASE)
AV: aortic valve
EACVI: European Association of Cardiovascular Imaging
EM: emergency medicine
ER: emergency room
ESICM: European Society of Intensive Care Medicine
FATE: Focused Assessed Transthoracic Echocardiography
HUD: handheld ultrasound device
IAC: Intersocietal Accreditation Commission for Echocardiography
ICU: intensive care unit
LV: left ventricular
ML: machine learning
MV: mitral valve
PHHE: pocket‐size handheld echocardiographic
PLAX: parasternal long axis
POC: point‐of‐care
POCUS: point‐of‐care ultrasound
PSAX: parasternal short axis
PUD: portable ultrasound device

## 1 Introduction

Ultrasound is a crucial tool for physicians in various specialties, offering rapid bedside diagnoses to address specific clinical cases. Among these, Focus‐Assessed Transthoracic Echocardiography (FATE) is a non‐invasive imaging test that uses ultrasound to assess the heart and major vessels, aiding in both ruling out differential diagnoses and confirming specific conditions [1]. Originally developed by Dr. Erik Sloth, a professor of point‐of‐care ultrasound (POCUS) and a consultant anesthetist from Denmark in 1989, FATE is a supplementary tool to physical examination [2]. Its ease of use and the advancements in ultrasound technology have made it increasingly popular worldwide, especially in scenarios requiring rapid diagnosis and reduced hospital stays [1].

In recent years, the use of ultrasound devices has become common in medical practice in addition to traditional physical examinations. These devices use high‐frequency sound waves to generate real‐time images of various body structures [3, 4]. The FATE protocol has gained global adoption due to its ability to facilitate rapid bedside diagnosis, its accessibility for non‐cardiologists, ease of learning, non‐ionizing radiation, non‐invasiveness, cloud‐sharing capabilities, telemedicine compatibility, and the portability of most devices [5]. Moreover, FATE devices offer advantages in terms of ease of cleaning, transportation, and disinfection compared to standard ultrasound machines [6]. While FATE shares similarities with other POCUS techniques in enabling the rapid diagnosis of critical conditions such as pleural effusions, cardiac tamponade, valvular dysfunction, and hemodynamic compromise, it surpasses them in its standardized and structured approach to cardiac assessment [7]. Unlike other POCUS methods, which may require operator‐dependent expertise for comprehensive cardiac evaluation, FATE provides a more systematic and reproducible framework, allowing for more consistent assessment of left ventricular (LV) function, volume status, and hemodynamic monitoring [7]. These advantages make FATE particularly valuable in resource‐limited settings and emergency situations where rapid, accurate cardiac evaluation is critical [7]. While FATE also shares similar functions with Focused Cardiac Ultrasound (FCU), it differentiates from FUC through its structured and standardized approach, providing a systematic assessment of global cardiac function, pericardial effusion, and volume status, whereas FCU is typically more limited and tailored to answering specific clinical questions. Also, while both are accessible to non‐cardiologists, FATE's structured nature enhances reproducibility across different operators, reducing the variability often seen in FCU, which is more operator dependent. Additionally, FATE extends beyond emergency and critical care settings, finding applications in perioperative monitoring, cardiology consultations, and telemedicine, making it a more versatile tool for comprehensive cardiac assessment [8].

A growing debate among physicians' centers around whether portable ultrasound devices (PUDs) will supersede the traditional stethoscope used for auscultation by enhancing bedside cardiac assessment by providing real‐time visualization of cardiac function, volume status, and hemodynamic parameters, allowing for more accurate diagnosis of conditions such as heart failure, valvular disease, and pericardial effusion—areas where auscultation alone may be limited [1]. However, the stethoscope remains a cost‐effective, rapid, and universally available tool, particularly in resource‐limited settings where ultrasound devices may not be accessible. Rather than being entirely replaced, the stethoscope will likely continue to play a complementary role, especially for initial screenings and in environments where affordability and ease of use are paramount.

The primary differences between FATE and comprehensive/limited (traditional) echocardiography include the level of expertise required for image acquisition and interpretation, the amount of information obtainable, and the scope of examination. While FATE can be conducted by non‐cardiologists, comprehensive/limited (traditional) echocardiography demands extensive training. The limited scope of FATE allows practitioners to leverage cardiac ultrasonography without the comprehensive training [often offered in structured courses from the American Society of Echocardiography (ASE) or the European Association of Cardiovascular Imaging (EACVI)] required for traditional echocardiography [7]. Notably, FATE does not replace the need for a full echocardiographic examination in all cases, but it provides valuable initial insights, particularly in time‐sensitive situations.

This narrative review explores the evolution and development of the FATE protocol, its current applications in various clinical scenarios, and its future prospects with advances in technology and greater equipment portability. Furthermore, the review discusses training requirements, image acquisition techniques, diagnostic accuracy, and interpretation pearls essential for proficient use of FATE. It also examines the growing evidence supporting its efficacy in trauma, critical care, preoperative assessment, emergency medicine, pre‐hospital settings, and resource‐limited environments. Additionally, the review highlights emerging technologies such as smaller devices that produce high‐quality image resolutions and innovations augmenting the capabilities of FATE. Finally, the review addresses its limitations and challenges, thereby paving the way for enhanced patient care and clinical outcomes.

## 2 Methods

This manuscript was conducted as a narrative review of the literature focusing on the development, methodology, clinical applications, training considerations, and emerging innovations related to Focused Assessed Transthoracic Echocardiography (FATE). A structured but non‐systematic literature search was performed using PubMed/MEDLINE, Google Scholar, and relevant professional society guidelines (including publications from the American Society of Echocardiography, European Association of Cardiovascular Imaging, American College of Emergency Physicians, and European Society of Intensive Care Medicine).

The literature search primarily included articles published from 1989 (the introduction of FATE) through 2024, with particular emphasis on landmark studies, consensus statements, clinical trials, and high‐impact reviews relevant to focused cardiac ultrasound and point‐of‐care (POC) echocardiography. Older foundational studies were included when necessary to provide historical context.

Studies were included based on their relevance to the conceptual framework, clinical utility, training requirements, diagnostic accuracy, technological advancements, and limitations of FATE. Both original research articles and authoritative review papers were considered. Case reports were included selectively when they provided unique clinical insights or illustrated novel applications of FATE. Non‐English publications, abstracts without full text, and studies lacking clear clinical relevance were excluded.

Given the narrative nature of this review, formal quality scoring and meta‐analytic techniques were not applied. Instead, evidence was synthesized qualitatively, with emphasis placed on consistency of findings, clinical applicability, and alignment with established guidelines and expert consensus.

## 3 Evolution of FATE

The origins of POCUS (including FATE) can be traced back to the mid‐20th century with the advent of echocardiography, pioneered by Inge Edler and Carl Hellmuth Hertz in the late 1950s [9]. Their groundbreaking work using ultrasound to visualize the heart laid the foundation for cardiac imaging techniques. Initially, echocardiography was cumbersome and time‐consuming, limited to specialized settings. However, the need for a rapid, non‐invasive, accurate method of assessing cardiac function and hemodynamics at the bedside, coupled with technological advances and the development of PUDs in the late 20th century, marked a turning point for echocardiography and led to the concept of FATE [10]. Like other POCUS protocols, FATE was designed to address specific clinical questions encountered in critical care settings, such as evaluating LV function, detecting pericardial effusions, assessing intravascular volume status, and identifying cardiac arrest. The goal was to provide timely, accurate information to guide clinical decision‐making and improve patient outcomes. Additionally, the development of handheld ultrasound devices (HUDs) with enhanced imaging capabilities has further integrated FATE into various clinical practices.

Since its introduction in 1989, FATE has evolved from a tool primarily used by cardiologists to one that can be readily learned by non‐cardiologists [11] and employed beyond traditional cardiology departments, such as in emergency rooms (ERs) [12], operating theaters, and intensive care units (ICUs) [12, 13]. The basic FATE protocol, proposed by Dr. Sloth in 2004, includes four fundamental views essential for cardiac assessment: the subcostal four‐chamber, apical four‐chamber (A4C), parasternal long axis (PLAX), parasternal short axis (PSAX), and pleural scanning views [2]. These views help rule out pleural pathologies and provide gross estimations of cardiac parameters such as chamber dimensions, wall thickness, and LV contractility. In 2010, the protocol was expanded to include extended apical and parasternal views along with Doppler ultrasound studies, termed the extended or advanced FATE protocol, offering a more comprehensive assessment of hemodynamics and valve function [2]. However, while FATE provides rapid bedside assessment, its measurements are less detailed compared to formal transthoracic echocardiography (TTE) guidelines, which include specific cutoffs for chamber dimensions, myocardial strain analysis, and precise Doppler‐derived hemodynamic parameters.

FATE assesses LV function primarily through qualitative “eyeball” estimation of LV contractility, visualizing endocardial motion in multiple views to approximate ejection fraction (EF). While this method is rapid and practical, it lacks the precision of quantitative assessments such as Simpson's biplane method or three‐dimensional echocardiography used in comprehensive TTE [2]. Additionally, FATE allows for indirect estimation of LV filling pressures using inferior vena cava (IVC) diameter and collapsibility, aiding in volume status assessment. Intravascular volume status is further evaluated using global cardiac size, IVC dynamics, and respiratory variation. These methods provide quick insights into preload and fluid responsiveness but are limited compared to invasive hemodynamic monitoring or more advanced Doppler‐based parameters like mitral inflow and tissue Doppler imaging (TDI) used in comprehensive echocardiography [2].

To enhance understanding, incorporating the FATE protocol card can serve as a visual guide for image acquisition, measurement techniques, and clinical interpretation. While FATE provides valuable bedside information, its proposed gross values must be interpreted with caution, as they do not always align with the normal reference ranges defined by major echocardiographic societies [2].

The adoption of FATE in medical education and clinical practice has been driven by its recognized utility and the efforts of professional organizations to standardize focused cardiac ultrasound training. Medical education curricula worldwide have incorporated basic POCUS techniques, including FATE, to equip students and healthcare providers with essential sonography skills [14]. Institutions such as the European Society of Intensive Care Medicine (ESICM) and the American College of Emergency Physicians (ACEP) offer specialized courses on focused cardiac ultrasound, which may include FATE as one of several standardized protocols. However, professional societies like the American Society of Echocardiography (ASE) and the European Association of Cardiovascular Imaging (EACVI) primarily emphasize comprehensive echocardiographic training rather than explicitly endorsing FATE [15, 16]. Nevertheless, the global adoption of focused cardiac ultrasound extends its reach to diverse healthcare settings, from remote clinics to urban hospitals, where its simplicity, cost‐effectiveness, and bedside applicability make it a valuable diagnostic tool.

## 4 Methodology of FATE

### 4.1 Training Requirements for Proficiency in POCUS (Including FATE)

Proficiency in FATE requires a strong understanding of cardiac anatomy, physiology, and ultrasound principles [8]. Healthcare providers seeking proficiency typically participate in structured training programs that include didactic lectures, hands‐on workshops, and supervised clinical practice [17, 18]. These programs vary in duration and intensity and cover essential topics such as ultrasound principles, physics, instrumentation, and knobology to optimize image quality during scanning. Additionally, trainees gain in‐depth knowledge of cardiac anatomy and physiology, including cardiac chambers, valves, great vessels, and blood flow dynamics to accurately interpret echocardiographic images [19, 20].

During training, individuals hone their image acquisition skills, including probe manipulation, patient positioning, and optimizing acoustic windows to capture clear and relevant echocardiographic views. Recognizing normal and abnormal findings is a core focus, with trainees learning to identify cardiac anatomy and common pathologies such as myocardial dysfunction, pericardial effusion, and valvular abnormalities for precise diagnosis [19, 20]. Trainees are also taught to integrate echocardiographic findings with clinical presentation, hemodynamic status, and other diagnostic modalities, enabling them to develop appropriate management strategies [21]. Quality assurance and continuing education are essential components, ensuring ongoing proficiency through feedback, peer review, and continuing medical education activities, thereby maintaining high standards of POCUS (including FATE) practice [14]. There are also echocardiography accreditation pathways in developed countries such as the British Society of Echocardiography (BSE) Level 1 Accreditation which was launched in April 2018 which serves as a foundational qualification for healthcare professionals aiming to perform focused echocardiographic evaluations in emergency settings [14]. The Focused Ultrasound in Intensive Care (FUSIC) which was developed by the Intensive Care Society also encompasses modules such as Heart, Lung, and Hemodynamics (HD), and provides clinicians with tools for comprehensive hemodynamic assessment using ultrasound, addressing key clinical questions related to cardiac, arterial, and venous structures [14]. Other accreditation programs include the European Association of Cardiovascular Imaging (EACVI) that guides echocardiography standards in Europe, and the Intersocietal Accreditation Commission (established in 1996) that accredits facilities in various echocardiographic modalities [14]. Table 1 below highlights the Training Requirements for Proficiency in FATE.

**Table 1:** Training requirements for proficiency in FATE.

Training component	Description	Goals
Didactic learning	Lectures and theoretical instruction	‐ Provides foundational knowledge of ultrasound principles and cardiac anatomy
Hands‐on workshops	Practical training with ultrasound devices	‐ Develops probe handling and image acquisition skills
Supervised clinical practice	Clinical scenarios under expert guidance	‐ Enhances real‐world application and interpretation proficiency
Quality assurance & feedback	Continuous assessment and peer review	‐ Maintains high standards and ensures competency over time
Continuing education	Ongoing learning and skill development	‐ Ensures up‐to‐date knowledge and incorporation of new techniques

### 4.2 Image Acquisition Techniques and Standard Views

Image acquisition in FATE involves obtaining specific standard views to systematically assess cardiac structures and function. Proper patient preparation is essential, with the patient positioned supine or semi‐recumbent with a slight left lateral tilt to optimize acoustic windows and facilitate image acquisition [3]. Selecting and placing the ultrasound probe strategically in various intercostal spaces is crucial for effectively visualizing different cardiac structures [2]. A phased‐array ultrasound probe is commonly used for its ability to provide both cardiac and lung views.

Standard views are then obtained to comprehensively evaluate the heart. The PLAX view offers a longitudinal plane of the LV, visualizing key structures such as the mitral valve (MV), LV outflow tract, and aortic valve (AV). The PSAX view, obtained at the level of the papillary muscles, demonstrates the LV chambers, papillary muscles, and MV in cross‐section. The A4C view displays all four cardiac chambers, assessing global systolic function, valvular function, and detecting intracardiac shunts. Additionally, the subxiphoid view allows visualization of the heart from a subcostal approach, particularly useful in patients with poor acoustic windows or inadequate parasternal views [2]. Dynamic assessment through real‐time imaging, with adjustments in probe position and angle, enables the evaluation of cardiac structure dimensions, wall motion abnormalities, and valvular function. Finally, image optimization techniques such as adjusting gain, depth, focus, and probe orientation are employed to enhance image quality and facilitate clear visualization of cardiac structures and possible pathologies [2]. Table 2 (FATE Standard Views and Their Diagnostic Relevance).

**Table 2:** FATE standard views and their diagnostic relevance.

Basic FATE views	Structures visualized	Diagnostic relevance
Subcostal four‐chamber	LA, LV, RA, RV, chamber wall thickness, IAS, IVS, MV, and TV	Chamber dilatation, hypertrophy, valvular dysfunction, atrial septal defect, pericardial effusion
Apical four‐chamber	LA, LV, RA, MV, TV, RV, IAS, and IVS	Ventricular dysfunction, dilatation, and hypertrophy, and pericardial effusion
Parasternal long‐axis	MV, AV, LA, LV and outflow tract, and descending aorta	LV systolic dysfunction, dilatation and hypertrophy of chambers, interventricular septal hypertrophy, mitral and aortic valvulopathy, descending aortic dilatation, pericardial and pleural effusion, ASD, VSD, TOF, and PDA
Parasternal short‐axis	Aorta, MV, papillary muscles, and LV chamber	Inferior Sinus Venosus Type of Atrial Septal Defects, pericardial effusion, valvular diseases, ASD, VSD, TOF, and PDA
Pleura scanning	Pleural and lungs	Pleural effusion, lung collapse or atelectasis, pleural thickening, pleural tumors, and pneumothorax

Abbreviations: ASD, atrial septal defect; AV, aortic valve; IAS, interatrial septum; IVS, interventricular septum; LA, left atrium; LV, left ventricle; MV, mitral valve; PDA, patent ductus arteriosus; RA, right atrium; RV, right ventricle; TOF, tetralogy of fallot; TV, tricuspid valve; VSD, ventricular septal defect.

### 4.3 Interpretation and Diagnostic Considerations in FATE

Interpreting FATE findings requires a systematic approach based on clinical data interpretation [21]. Key diagnostic considerations encompass various aspects of cardiac function and pathology. Assessing global systolic function, particularly LV contractility, is pivotal. This evaluation employs both qualitative methods such as visual estimation and quantitative methods such as calculating the EF, aiding in diagnosing and managing cardiac dysfunction effectively.

Evaluating valvular function involves scrutinizing valvular morphology, mobility, and hemodynamic significance to provide insights into conditions like valvular heart disease, including stenosis, regurgitation, and endocarditis. Pericardial assessment is crucial for diagnosing conditions such as pericardial effusion. Detecting effusions and assessing their size, distribution, and hemodynamic impact are imperative, especially in patients suspected of having pericardial tamponade or effusive‐constrictive physiology.

Additionally, evaluating volume status is essential for guiding fluid management in critically ill patients. Parameters such as IVC size and collapsibility offer valuable information about intravascular volume responsiveness, aiding clinicians in making informed decisions regarding fluid therapy [17]. Lastly, integrating echocardiographic findings with the patient's clinical presentation, hemodynamic parameters, and other diagnostic tests is vital. This correlation facilitates accurate diagnosis and guides appropriate therapeutic interventions tailored to the individual patient's needs [3, 21].

## 5 Clinical Applications of FATE

FATE is recognized for its efficiency, non‐invasive approach, and ease of repeatability as needed, making it a valuable diagnostic method. It helps identify the causes of hypotension and shock, facilitating critical interventions [22]. In the initial evaluation of trauma patients, FATE provides important findings that helps identify life‐threatening conditions such as pericardial effusion, cardiac tamponade, and pneumothorax [23, 24], expediting diagnosis and guiding urgent interventions. This results in enhanced patient outcomes and reduced mortality rates through early detection and management of these life‐threatening conditions. This is backed by a recent study which suggests that pre‐operative focused TTE (such as FATE) is associated with lower mortality in patients at an increased risk of cardiac risk undergoing femoral neck fracture surgery compared to those who did not undergo focus TTE [25].

In critically ill patients, FATE provides a focused approach to echocardiographic examination, offering a skill set that can be readily applied [26]. It serves as a valuable tool for hemodynamic monitoring, cardiac function assessment, and the identification of acute cardiovascular abnormalities in these patients [26]. Its real‐time imaging capabilities enable swift decision‐making in managing conditions such as sepsis, shock (hypovolemic, cardiogenic, obstructive), or respiratory failure due to chronic lung diseases, pulmonary embolism, and heart failure [27].

FATE‐guided interventions have been shown to improve fluid resuscitation strategies and optimize hemodynamic stability in ICU and ER settings [28] by providing dynamic assessments of intravascular volume and cardiac function. Specifically, FATE allows for the evaluation of the IVC diameter and collapsibility, helping to determine fluid responsiveness [23]. A highly collapsible IVC suggests volume depletion and the need for fluid resuscitation, whereas a distended, non‐collapsible IVC may indicate fluid overload or elevated right atrial pressures [23]. Additionally, FATE assesses LV function and cardiac output, aiding in distinguishing different shock states and guiding targeted interventions. In patients with sepsis, for example, FATE can help differentiate between hypovolemia and myocardial dysfunction, ensuring appropriate use of fluids, vasopressors, or inotropes [26]. By integrating these echocardiographic findings, clinicians can tailor fluid management strategies to prevent both under‐resuscitation, which may lead to organ hypoperfusion, and over‐resuscitation, which can cause pulmonary edema and worsening heart failure [23].

FATE is pivotal in preoperative evaluation; it aids in assessing the risk of perioperative cardiac complications in non‐cardiac surgeries [29]. In a study by Kratz et al., Intraoperative focused echocardiography was performed in all 50 patients experiencing hemodynamic instability. In 33 cases (66%, 95% CI: 52.11–77.61), the findings from focused echocardiogram directly influenced clinical management [29]. A total of 82 episodes of hemodynamic instability were documented, with focused‐echo prompting a change in treatment in 38 instances (46.34%, 95% CI: 35.95–57.06). The most frequently identified condition was hypovolemia, observed in 66% of cases, while right‐heart overload was present in 22%, and right‐heart failure was detected in 4% of patients [29]. Perioperative FATE assists in diagnosing new cardiac conditions for anesthesia management, intraoperative monitoring, hemodynamic therapy, and postoperative care, potentially preventing procedural delays and unnecessary consultations [30]. FATE findings can inform perioperative management decisions such as fluid optimization strategies and anesthesia technique selection, ultimately contributing to improved surgical outcomes and patient safety [31, 32].

FATE serves as an excellent POC test due to its portability and simplicity, making it suitable for use in resource‐limited environments. In these settings with restricted access to advanced imaging modalities, it serves as a valuable diagnostic tool for healthcare providers [33, 34]. Its user‐friendly interface and rapid imaging capabilities enable timely assessment and management of patients in field hospitals, disaster response scenarios, and remote healthcare settings [35]. Table 3 (overview of FATE applications and clinical benefits).

**Table 3:** Overview of Focused Assessed Transthoracic Echocardiography (FATE) applications and clinical benefits.

Clinical setting	Applications of FATE	Clinical benefits
Trauma	‐ Identify pericardial effusion and tamponade	‐ Enables rapid diagnosis and critical interventions
	‐ Assess cardiac injury (e.g., contusions)	‐ Guides emergent surgical or procedural decisions
Critical care	‐ Evaluate left ventricular function	‐ Provides hemodynamic monitoring in real‐time
	‐ Assess fluid responsiveness	‐ Guides fluid therapy and vasopressor management
	‐ Identify acute cardiovascular abnormalities	‐ Facilitates early intervention and improves outcomes
Preoperative	‐ Assess cardiac risk in non‐cardiac surgeries	‐ Supports anesthesia management and perioperative care
	‐ Identify new cardiac pathologies	‐ Reduces procedural delays and unnecessary consults
Emergency medicine	‐ Assess cardiac function in shock and cardiac arrest	‐ Guides immediate treatment and resuscitation strategies
	‐ Identify cardiac causes of chest pain	‐ Speeds up triage and decision‐making in emergency settings
Resource‐limited environments	‐ Provide bedside cardiac imaging	‐ Offers cost‐effective and portable diagnostic tool
	‐ Facilitate remote patient assessment	‐ Reduces need for advanced imaging resources

## 6 Evidence Supporting the Efficacy of FATE

Clinical studies (including observational and prospective cohort studies) and trials consistently affirm FATE's effectiveness in rapidly evaluating cardiac function and detecting abnormalities. FATE has demonstrated high sensitivity and specificity in diagnosing conditions such as pericardial effusion, cardiac tamponade, and acute myocardial infarction [36, 37, 38]. Numerous studies have explored FATE's efficacy in various clinical contexts. For instance, observational studies have shown fluid responsiveness can be accurately predicted using the collapsibility index of the IVC, which can be assessed with FATE; thus, providing strong evidence of its benefits in this regard [39, 40]. Other studies including prospective cohort studies have also demonstrated that FATE‐based recommendations improved survival rates, patient outcomes, and reduced complications [28, 30, 41].

FATE has been shown to impact diagnosis and management in ICUs [42]. It can identify unexpected pathology in patients undergoing urgent surgical procedures, leading to changes in anesthesia techniques or supportive actions [43]. By enabling early diagnosis and timely intervention, FATE enhances clinical decision‐making and management strategies, leading to expedited treatment and improved overall prognosis for patients [44].

Overall while high‐quality evidence from prospective cohort studies and guideline‐based recommendations supports the role of FATE in the management decision and improving perioperative and critical care workflows, definitive outcome data remain limited. The variability in training standards, image acquisition. Protocols, and outcome measures across different studies underscores the need for larger prospective trials and standardized competency frameworks. These limitations should temper overgeneralization of findings while reinforcing the value of FATE as a complementary rather than replacement tool to comprehensive echocardiography. Table 4 provides a summary table including major studies focusing on FATE.

**Table 4:** Key studies evaluating Focused Assessed Transthoracic Echocardiography (FATE).

Author/Year	Study design	Clinical setting	Primary focus	Key findings	Strength of evidence	Limitations	Relevance to FATE
Sloth et al., 2004	Descriptive protocol	Perioperative/ICU	FATE protocol development	Defined standardized focused cardiac views for rapid bedside assessment	Foundational	No outcome data	Established FATE framework
Via et al., 2014	International guideline	ICU/Emergency	Focused cardiac ultrasound standards	Endorsed focused echo for rapid clinical decision‐making	High	Not FATE‐exclusive	Supports FATE principles
Kanji et al., 2014	Prospective cohort	ICU (shock)	Echo‐guided management	Altered therapy and improved outcomes	Moderate–High	Single‐center	Validates focused echo utility
Canty et al., 2012	Retrospective cohort	Preoperative	Focused TTE outcomes	Lower mortality in high‐risk surgical patients	Moderate	Retrospective	Indirect support for FATE
Kratz et al., 2017	Prospective observational	Intraoperative	Hemodynamic instability	Management changed in ~66% of cases	Moderate	Small sample size	Supports intraoperative FATE
Huson et al., 2019	Observational program	Resource‐limited settings	Feasibility of focused echo	Demonstrated feasibility and diagnostic value	Low–Moderate	No outcome endpoints	Supports global FATE use
Zhang et al., 2018	Prospective AI validation	Multicenter	AI‐assisted echocardiography	Improved reproducibility and accuracy	High	Not FATE‐specific	Relevant to future FATE integration

## 7 Emerging Technologies and Innovations in FATE

While the following are not entirely specific to FATE, they are emerging technologies and innovations in POCUS (including FATE).

### 7.1 Integration of Artificial Intelligence (AI) and Machine Learning (ML) in FATE Interpretation

The integration of AI and ML in FATE interpretation is an emerging area of focus in medicine, aiming to address inconsistencies and variability in image acquisition and interpretation [45]. Unlike other imaging modalities such as computed tomography or magnetic resonance imaging, echocardiography often experiences interobserver variability and relies on the operator's experience level. Given the increasing demand and complexity in cardiovascular imaging, reducing variability among echocardiographers, and enhancing efficiency are essential goals. AI shows promise in this regard, potentially extracting information not readily discernible to the human eye and overcoming limitations such as fatigue, distraction, and human variability [46]. ML has already demonstrated potential in diagnosing structural heart disease and assessing cardiac landmarks for LV function and volumes [47]. Additionally, AI assists with image acquisition by offering automated measurement features and image optimization, thereby saving time and standardizing results [47]. It is however key to note that despite these benefits, the integration of AI into FATE warrants rigorous validation through prospective multicenter studies to assess diagnostic accuracy, clinical impact, and generalization especially across diverse population. Future work should also address the regulatory considerations such as the alignment with established medical device approval pathways, transparency of algorithm training data, and the safeguards for clinical accountability when AI‐assisted interpretations are used.

### 7.2 Advancements in Handheld Ultrasound Devices in Enhancing Portability and Accessibility

Advancements in HUDs have revolutionized cardiac imaging by enhancing portability and accessibility. Traditional echocardiography often relies on high‐end systems in specialized echo laboratories, which can be bulky and expensive. However, the advent of pocket‐size handheld echocardiographic (PHHE) devices has transformed this landscape, enabling POC echocardiography with reduced cost and increased accessibility [48]. PHHE devices offer rapid acquisition (under 5 min) and accurate detection of cardiac pathologies, facilitating early diagnosis and intervention in emergency settings [49]. Several studies have demonstrated a good correlation between echocardiographic findings from PHHE and standard echocardiography, supporting their effectiveness. Continued development of affordable and easy‐to‐use PHHE with increased scope and efficiency will further increase the global adoption of FATE.

### 7.3 Novel Applications and Future Directions of FATE

The integration of emerging technologies opens endless possibilities for innovative uses of FATE. One innovative application is its integration with telemedicine platforms. As telemedicine becomes increasingly popular in remote and underserved areas, FATE can serve as a valuable tool for real‐time assessment and monitoring of various pathologies. By utilizing HUDs and telecommunication, healthcare providers can remotely perform FATE examinations, allowing for timely diagnosis and management of various medical conditions without the need for in‐person visits. This application holds significant promise for extending medical care to regions with limited access to specialized medical facilities, thereby improving patient outcomes and reducing healthcare disparities.

Another innovative application of FATE involves its integration with wearable devices and smart technology. Pairing FATE with wearable cardiac monitoring devices, such as smartwatches or patches, allows individuals to perform self‐assessments of their cardiac health in real‐time. This approach enables continuous monitoring of cardiac function and early detection of abnormalities, empowering individuals to proactively manage their cardiovascular health. Additionally, data obtained from these wearable devices can be transmitted to healthcare providers for remote interpretation and intervention, facilitating personalized and preventive care strategies. This novel use of FATE in conjunction with wearable technology has the potential to revolutionize cardiac monitoring and management, promoting early detection and intervention for improved patient outcomes.

**Figure 1:**
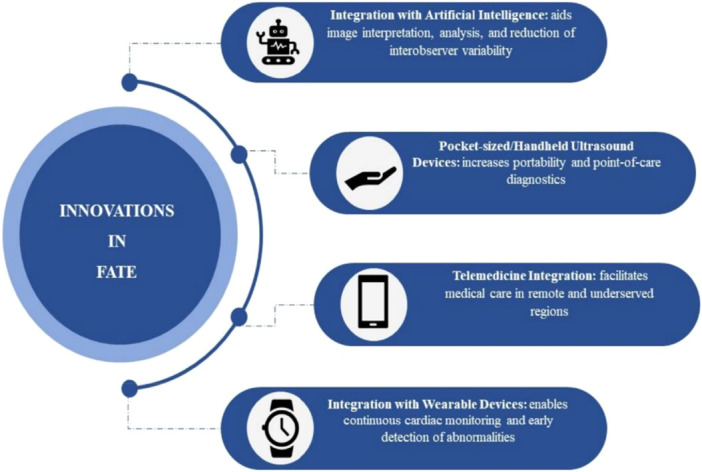
Emerging technologies and innovations in Focused Assessment Transthoracic Echocardiography (FATE).

Finally, advancements in technology will further improve the quality and interpretation of images obtained from FATE, ensuring its continued relevance and effectiveness in clinical practice [45]. Figure 1 (Emerging technologies and innovations in FATE).

## 8 Challenges and Limitations of FATE

### 8.1 Operator‐Dependent Variability and Skill Retention

Operator‐dependent variability and skill retention are critical considerations in the effective implementation of FATE. While FATE is increasingly recognized as a vital clinical decision‐making tool, its optimal utilization requires a certain level of proficiency to avoid potential errors that could negatively impact patient outcomes. Inexperienced operators may inadvertently misdiagnose conditions, leading to inappropriate treatment decisions or, in severe cases, fatal consequences [44]. Additionally, the subjective nature of cardiac ultrasound interpretation and its reliance on operator expertise introduces challenges, with experienced clinicians sometimes showing discrepancies in their interpretations, especially concerning findings like LV wall motion abnormalities [50]. To mitigate these challenges and enhance operator accuracy, comprehensive training programs and standardized competency assessment methods are crucial. Research suggests that achieving competence in image acquisition and interpretation demands substantial practice and ongoing mentorship from seasoned clinicians [51].

### 8.2 Quality Assurance and Standardization Issues

Ensuring quality assurance and standardization is essential for maintaining the integrity of FATE. Adequate patient data collection and clear indication for performing echocardiographic studies are important for facilitating auditing, traceability, and root cause analysis of potential errors in image acquisition and interpretation. However, casual utilization of FATE without a defined indication poses a significant challenge to quality assurance efforts. Standardizing FATE protocols and adhering to established guidelines are fundamental for ensuring consistency and accuracy across interpretations. Continuing education programs accredited by recognized bodies such as the Intersocietal Accreditation Commission for Echocardiography (IAC) are vital for equipping healthcare providers with updated knowledge and ensuring compliance with standardized protocols [52].

### 8.3 Addressing Barriers to Widespread Adoption and Acceptance of FATE

**Figure 2:**
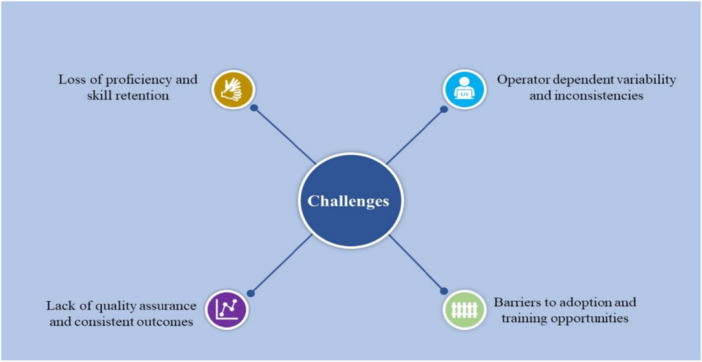
Challenges and limitations of FATE.

Despite the many benefits of FATE, barriers to its widespread adoption persist, such as inadequate training opportunities within residency programs and a shortage of proficient trainers. A 2020 multicenter survey across the United States and Canada identified lack of training and HUDs as top barriers to the widespread adoption of POCUS, followed closely by the lack of direct supervision and time to perform POCUS during rounds [52]. Addressing these barriers requires concerted efforts to integrate comprehensive FATE training into medical curriculums and provide sufficient resources for ongoing education and mentorship [14]. Initiatives such as providing HUDs to medical students and residents, along with robust training programs, standardized competency metrics and training completion certificates, exemplify promising steps toward overcoming these barriers and promoting the widespread acceptance and utilization of FATE [53]. Figure 2 (Challenges and limitations of FATE).

Addressing these concerns will be key to safely adopting AI integration into FATE and maintaining high standards of patient care. Addressing these research priorities will ultimately help transition FATE from predominantly operator dependent to a standardized technologically augmented modality capable of delivery consistent, high quality cardiac assessment across healthcare systems.

## 9 Conclusion

In conclusion, this narrative review summarized the evolution, methodology, clinical applications, evidence supporting efficacy, emerging technologies, and challenges surrounding the utilization of FATE. It highlighted FATE's pivotal role in modern medicine as a rapid, non‐invasive diagnostic tool that aids in assessing cardiac function and hemodynamics at the bedside, particularly in acute care settings. With its portability, ease of use, and ability to provide real‐time imaging, FATE has become increasingly indispensable across various medical specialties, including emergency medicine, critical care, anesthesia, and preoperative assessment. The review emphasized the importance of structured training programs to ensure proficiency among healthcare providers and addressed ongoing efforts to overcome barriers to widespread adoption, such as inadequate training opportunities and resource constraints.

The implications of FATE for modern medicine and patient care are profound. Its ability to provide timely and accurate diagnostic information empowers clinicians to make informed decisions, leading to improved patient outcomes and reduced morbidity and mortality rates. FATE's versatility and adaptability make it well‐suited for diverse clinical scenarios, ranging from rapid evaluation of critically ill patients to preoperative assessment and monitoring in resource‐limited settings. Additionally, the integration of FATE with emerging technologies such as AI and wearable devices holds promise for further enhancing its diagnostic capabilities and expanding its utility in remote healthcare delivery and personalized medicine.

Looking ahead, future research in the field of FATE should include efforts to standardize protocols, address operator‐dependent variability, and optimize training methodologies. Further exploration of FATE's role in telemedicine, remote monitoring, and preventive care represents fertile ground for research, with potential implications for healthcare delivery and patient outcomes on a global scale. Continued innovation in handheld ultrasound devices and advancements in image processing techniques will contribute to improving the accessibility, accuracy, and efficiency of FATE. Overall, the ongoing evolution and integration of FATE into clinical practice highlights its significance as a valuable tool for enhancing patient care and advancing the field of diagnostic medicine.

## Author Contributions

**Habib Olatunji Alagbo:** conceptualization, methodology, literature search, writing – original draft, writing – review and editing. **Oluwaremilekun Zeth Tolu‐Akinnawo:** conceptualization, methodology, investigation, literature search, data curation, formal analysis, validation, visualization, writing – original draft, writing – review and editing, supervision, project administration, resources. **Isaac Adewumi Babawale:** literature search, data curation, writing – review and editing. **Charles Oluwarotimi Poluyi:** writing – review and editing, validation. **Selimat Ibrahim:** literature search, writing – review and editing. **Jonas Lotanna Ibekwe:** literature search, writing – review and editing. **Toluwalase Awoyemi:** supervision, conceptualization, methodology, validation, writing – review and editing, project administration.

## Disclosure

All authors have read and approved the final version of the manuscript. The CORRESPONDING AUTHOR; had full access to all of the data in this study and takes complete responsibility for the integrity of the data and the accuracy of the data analysis.

## Conflicts of Interest

The authors declare no conflicts of interest.

## Transparency Statement

The lead author Toluwalase Awoyemi affirms that this manuscript is an honest, accurate, and transparent account of the study being reported; that no important aspects of the study have been omitted; and that any discrepancies from the study as planned (and, if relevant, registered) have been explained.

## Data Availability

The data that support the findings of this study are available from the corresponding author upon reasonable request.
